# A global database of soil microbial phospholipid fatty acids and enzyme activities

**DOI:** 10.1038/s41597-025-05759-2

**Published:** 2025-09-26

**Authors:** Laura G. van Galen, Gabriel Reuben Smith, Andrew J. Margenot, Mark P. Waldrop, Thomas W. Crowther, Kabir G. Peay, Robert B. Jackson, Kailiang Yu, Anna Abrahão, Talaat A. Ahmed, Juha M. Alatalo, Sten Anslan, Mark A. Anthony, Ademir Sergio Ferreira Araujo, Judith Ascher-Jenull, Elizabeth M. Bach, Mohammad Bahram, Christopher C. M. Baker, Petr Baldrian, Richard D. Bardgett, M. Noelia Barrios-Garcia, Felipe Bastida, Francesca Beggi, Liane G. Benning, Luca Bragazza, Arthur A. D. Broadbent, Concha Cano-Díaz, Anna M. Cates, Carlos E. P. Cerri, Simone Cesarz, Baodong Chen, Aimeé T. Classen, Mathilde Borg Dahl, Manuel Delgado-Baquerizo, Nico Eisenhauer, Svetlana Yu. Evgrafova, Nicolas Fanin, Flavio Fornasier, Romeu Francisco, André L. C. Franco, Serita D. Frey, Hannu Fritze, Carlos García, Pablo García-Palacios, María Gómez-Brandón, Marina Gonzalez-Polo, Beatriz Gozalo, Robert Griffiths, Carlos Guerra, Moritz Hallama, Inga Hiiesalu, Mohammad Zabed Hossain, Yajun Hu, Heribert Insam, Vincent E. J. Jassey, Lili Jiang, Ellen Kandeler, Petr Kohout, Urmas Kõljalg, Valentyna Krashevska, Xiaofei Li, Jing-Zhong Lu, Xiankai Lu, Shan Luo, Stefanie Lutz, Kathleen Allison Mackie-Haas, Fernando T. Maestre, Minna Malmivaara-Lämsä, Kai Mangelsdorf, Maria Manjarrez, Sven Marhan, Ashley Martin, Kelly E. Mason, Jordan Mayor, Rebecca L. McCulley, Mari Moora, Paula V. Morais, Miriam Muñoz-Rojas, Rajasekaran Murugan, Andrew T. Nottingham, Victoria Ochoa, Raúl Ochoa-Hueso, Jane Oja, Pål Axel Olsson, Maarja Öpik, Nick Ostle, Krista Peltoniemi, Taina Pennanen, David S. Pescador, G. Kenny Png, Christian Poll, Sergei Põlme, Anton M. Potapov, Anders Priemé, William Pritchard, Jeremy Puissant, Sandra Mara Barbosa Rocha, Christoph Rosinger, Liliane Ruess, Emma J. Sayer, Stefan Scheu, Robert L. Sinsabaugh, Lindsey C. Slaughter, Nadejda A. Soudzilovskaia, José Paulo Sousa, Lee Stanish, Shu-ichi Sugiyama, Leho Tedersoo, Pankaj Trivedi, Tanel Vahter, Jana Voriskova, Dirk Wagner, Cong Wang, David A. Wardle, Jeanette Whitaker, Yuanhe Yang, Zhiwei Zhong, Kai Zhu, Lori A. Ziolkowski, Martin Zobel, Johan van den Hoogen

**Affiliations:** 1 Institute of Integrative Biology, Department of Environmental Systems Sciences, ETH Zurich, Zurich, Switzerland; 2 Department of Biology, Stanford University, Stanford, California USA; 3 Department of Crop Sciences, University of Illinois Urbana-Champaign, Urbana, IL 61801 USA; 4 Agroecosystem Sustainability Center (ASC), Institute for Sustainability, Energy and Environment (iSEE), Urbana, IL 61801 USA; 5 US Geological Survey, Geology, Minerals, Energy, and Geophysics Science Center, Menlo Park, California USA; 6 Department of Earth System Science, Stanford University, Stanford, California USA; 7 Department of Ecology and Evolutionary Biology, Princeton University, Princeton, NJ USA; 8 High Meadows Environmental Institute, Princeton University, Princeton, NJ USA; 9 Department of Biology, Science Center, Federal University of Ceará – UFC, Fortaleza, CE 60440-900 Brazil; 10 Environmental Science Center, Qatar University, Doha, Qatar; 11 Department of Biological and Environmental Science, University of Jyväskylä, Jyväskylä, Finland; 12 Department of Biology, College of Science, Princess Nourah bint Abdulrahman University, Riyadh, Saudi Arabia; 13 Center for Microbiology and Environmental Systems Science, University of Vienna, 1030 Vienna, Austria; 14 Soil Microbial Ecology Group, Universidade Federal do Piauí (UFPI), 64019-550 Teresina, Piauí Brazil; 15 Department of Experimental Architecture, Integrative Design Extremes, University of Innsbruck, Technikerstrasse 21, Innsbruck, A-6020 Austria; 16 The Nature Conservancy in Illinois, Nachusa Grasslands, Franklin Grove, IL USA; 17 Department of Ecology, Swedish University of Agricultural Sciences, Uppsala, Ulls väg 16, 756 51 Sweden; 18 Department of Agroecology, Aarhus University, Slagelse, 4200 Denmark; 19 US Army ERDC Cold Regions Research and Engineering Laboratory, Hanover, NH 03755 USA; 20 Laboratory of Environmental Microbiology, Institute of Microbiology of the Czech Academy of Sciences, Videnska 1083, 14200 Praha 4, Prague, Czech Republic; 21 Department of Earth and Environmental Sciences, The University of Manchester, Manchester, M13 9PT UK; 22 Lancaster Environment Centre, Lancaster University, Lancaster, LA1 4YQ UK; 23 Rubenstein School of Environment and Natural Resources, University of Vermont, Burlington, VT USA; 24 CENAC-APN, CONICET, Bariloche, Argentina; 25 CEBAS-CSIC Department of Soil and Water Conservation Campus Universitario de Espinardo, 30100 Murcia, Spain; 26 Alliance Bioversity International - CIAT, Bengaluru, India; 27 GFZ Helmholtz Centre for Geosciences, Interface Geochemistry Section, Telegrafenberg, 14473 Potsdam, Germany; 28 Department of Earth Sciences, Freie Universität Berlin, 12249 Berlin, Germany; 29 Field-Crop Systems and Plant Nutrition, Agroscope, Route de Duillier 60, CH-1260 Nyon, Switzerland; 30 Biological & Environmental Sciences, Faculty of Natural Sciences, University of Stirling, FK9 4LA Stirling, UK; 31 CISAS - Centre for Research and Development in Agrifood Systems and Sustainability, Polytechnic Institute of Viana do Castelo, Rua Escola Industrial e Comercial Nun’Álvares, 34, 4900-347 Viana do Castelo, Portugal; 32 Department of Soil, Water, and Climate, University of Minnesota, 1991 Upper Buford Circle, St Paul, MN 55108 USA; 33 University of São Paulo ESALQ/USP Av Pádua Dias 11, 13418-900 Piracicaba, SP Brazil; 34 German Centre for Integrative Biodiversity Research (iDiv) Halle-Jena-Leipzig, Puschstrasse 4, 04103 Leipzig, Germany; 35 Institute of Biology, Leipzig University, Puschstrasse 4, 04103 Leipzig, Germany; 36 State Key Laboratory of Regional and Urban Ecology, Research Center for Eco-Environmental Sciences, Chinese Academy of Sciences, Beijing, China; 37 College of Resources and Environment, University of Chinese Academy of Sciences, Beijing, China; 38 Ecology and Evolutionary Biology Department, University of Michigan, Ann Arbor, MI 48109 USA; 39 Institute of Microbiology, Center for Functional Genomics of Microbes, University of Greifswald, 17489 Greifswald, Germany; 40 Laboratorio de Biodiversidad y Funcionamiento Ecosistémico, Instituto de Recursos Naturales y Agrobiología de Sevilla (IRNAS), Consejo Superior de Investigaciones Científicas (CSIC), Av., Reina Mercedes 10, E-41012 Sevilla, Spain; 41 Sukachev Institute of Forest SB RAS, Federal Research Center “Krasnoyarsk Science Center SB RAS”, Krasnoyarsk, 660036 Russia; 42 Melnikov Permafrost Institute, Siberian Branch, Russian Academy of Sciences, 677010 Yakutsk, Russia; 43 INRAE, Bordeaux Sciences Agro, UMR 1391 Interaction Soil Plant Atmosphere (ISPA), 71 avenue Edouard Bourlaux, 33882 Villenave-d’Ornon cedex, France; 44 CREA Research Center for Viticulture and Enology, Gorizia, Italy; 45 University of Coimbra, Centre for Mechanical Engineering, Materials and Processes, ARISE, Department of Life Sciences, 3000-456 Coimbra, Portugal; 46 O’Neill School of Public and Environmental Affairs, Indiana University, Bloomington, IN 47405 USA; 47 Department of Natural Resources and the Environment, University of New Hampshire, Durham, NH 03824 USA; 48 Natural Resources Institute Finland, Luke, Latokartanonkaari 9, 00790 Helsinki, Finland; 49 Instituto de Ciencias Agrarias, Consejo Superior de Investigaciones Científicas, Serrano 115 bis, 28006 Madrid, Spain; 50 Animal Ecology Group (GEA), University of Vigo, E-36310 Vigo, Spain; 51 INIBIOMA, CONICET- Universidad Nacional del Comahue, Bariloche, Argentina; 52 Instituto Multidisciplinar para el Estudio del Medio “Ramón Margalef”, Universidad de Alicante, Alicante, Spain; 53 Departamento de Ecología, Universidad de Alicante, Alicante, Spain; 54 School of Environmental and Natural Sciences, Bangor University, Gwynedd, LL57 2DG UK; 55 University of Coimbra, Department of Geography, Colégio de São Jerónimo, Largo D.Dinis, 3004-530 Coimbra, Portugal; 56 Institute of Soil Science and Land Evaluation, University of Hohenheim, D-70593 Stuttgart-Hohenheim, Germany; 57 Institute of Ecology and Earth Sciences, University of Tartu, 2 J. Liivi St, 50409 Tartu, Estonia; 58 Department of Botany, University of Dhaka, Dhaka, 1000 Bangladesh; 59 College of Agronomy, Hunan Agricultural University, Changsha, 410128 P.R. China; 60 BioTreaT GmbH, Technikerstrasse 21, 6020 Innsbruck, Austria; 61 Centre de Recherche sur la Biodiversité et l’Environnement (CRBE), Université de Toulouse, CNRS, IRD, Toulouse INP, Université Toulouse 3 – Paul Sabatier (UT3), Toulouse, France; 62 State Key Laboratory of Tibetan Plateau Earth System, Environment and Resources (TPESER), Institute of Tibetan Plateau Research, Chinese Academy of Sciences, Beijing, 100101 China; 63 Laboratory of Microbial Ecology and Biogeography, Institute of Microbiology of the Czech Academy of Sciences, Videnska 1083, 14200 Praha 4, Prague, Czech Republic; 64 Senckenberg Biodiversity and Climate Research Centre, Functional Environmental Genomics, Frankfurt, Germany; 65 College of Resources and Environmental Sciences/Key Laboratory of Sustainable Utilization of Soil Resources in the Commodity Grain Bases in Jilin Province, Jilin Agricultural University, Changchun, 130118 China; 66 Johann Friedrich Blumenbach Institute of Zoology and Anthropology, University of Göttingen, Untere Karspüle 2, 37073 Göttingen, Germany; 67 Key Laboratory of Vegetation Restoration and Management of Degraded Ecosystems, South China Botanical Garden, Chinese Academy of Sciences, Guangzhou, China; 68 Department of Evolution, Ecology, and Behaviour, University of Liverpool, Crown street, Liverpool, L69 7BE UK; 69 Plant-Soil Interactions, Agroecology and Environment, Agroscope, Reckenholzstrasse 191, 8046 Zurich, Switzerland; 70 Agroscope, Viticulture, 8820 Wädenswil, Switzerland; 71 Environmental Sciences and Engineering, Biological and Environmental Science and Engineering Division, King Abdullah University of Science and Technology, Thuwal, 23955-6900 Kingdom of Saudi Arabia; 72 GFZ Helmholtz Centre for Geosciences, Organic Geochemistry Section, Telegrafenberg, 14473 Potsdam, Germany; 73 Microbiology Laboratories Australia, St Marys, 5042 Australia; 74 Microbiology Laboratories Australia, Adelaide, Australia; 75 Benevita Biotech, Adelaide, Australia; 76 UK Centre for Ecology & Hydrology, Library Avenue, Bailrigg, Lancaster, LA1 4AP UK; 77 ICF, 201 Mission St. Suite 1500, San Francisco, CA 94105 USA; 78 Department of Plant & Soil Sciences, University of Kentucky, Lexington, KY 40546-0312 USA; 79 Centre for Ecosystem Science, School of Biological, Earth & Environmental Sciences, UNSW, Sydney, 2052 NSW Australia; 80 Valli Sustainability Research and Education Foundation, Kanchipuram, India; 81 Institute of Soil Research, Department of Ecosystem Management, Climate and Biodiversity, BOKU University, 1190 Vienna, Austria; 82 School of Geography, University of Leeds, Leeds, LS2 9JT UK; 83 Instituto Univeritario de Investigación en Olivar y Aceite de Oliva-INUO, Universidad de Jaén, Jaén, Spain; 84 Department of Biology, IVAGRO, University of Cádiz, Campus de Excelencia Internacional Agroalimentario (CeiA3), Campus del Rio San Pedro, 11510 Puerto Real, Cádiz, Spain; 85 Department of Terrestrial Ecology, Netherlands Institute of Ecology (NIOO-KNAW), P.O. Box 50, 6700 AB Wageningen, the Netherlands; 86 Department of Biology, Lund University, SE-223 62 Lund, Sweden; 87 Departamento de Farmacología, Farmacognosia y Botánica, Facultad de Farmacia, Universidad Complutense de Madrid, Madrid, Spain, Pl. de Ramón y Cajal, s/n, Moncloa - Aravaca, 28040 Madrid, Spain; 88 Singapore Botanic Gardens, National Parks Board, Singapore, 259569 Republic of Singapore; 89 Natural History Museum of Tartu University, 46 Vanemuise St., 51014 Tartu, Estonia; 90 Senckenberg Museum of Natural History Gorlitz, Am Museum 1, D-02826 Görlitz, Germany; 91 Department of Biology and Center for Volatile Interactions (VOLT), University of Copenhagen, Copenhagen, Denmark; 92 Laboratoire d’Ecologie Alpine, Univ. Grenoble Alpes, Univ. Savoie Mont Blanc, CNRS, LECA, Grenoble, France; 93 Institute of Agronomy, Department of Agricultural Sciences, BOKU University, 3430 Tulln an der Donau, Austria; 94 Institute of Biology, Ecology Group, Humboldt-Universität zu Berlin, Philippstraße 13, 10115 Berlin, Germany; 95 Institute of Botany, Ulm University, 89081 Ulm, Germany; 96 Centre of Biodiversity and Sustainable Land Use, University of Göttingen, Büsgenweg 1, 37077 Göttingen, Germany; 97 Biology Department, University of New Mexico, Albuquerque, NM 87131 USA; 98 Texas Tech University, Department of Plant and Soil Science, Lubbock, TX 79409 USA; 99 Centre for Environmental Sciences, Hasselt University - Campus Diepenbeek Agoralaan Building D, Units AB, B-3590 Diepenbeek, Belgium; 100 Centre for Functional Ecology, Associate Laboratory TERRA, Department of Life Sciences, University of Coimbra, 3000-456 Coimbra, Portugal; 101 Institute of Arctic and Alpine Research University of Colorado, Boulder, CO USA; 102 Faculty of Agriculture and Life Science, Hirosaki University, Bunkyocho 1, Hirosaki 036-8560, Aomori, Japan; 103 Mycology and Microbiology Center, University of Tartu, Tartu, Estonia; 104 Department of Zoology, College of Science, King Saud University, Riyadh, Saudi Arabia; 105 Microbiome Network and Department of Agricultural Biology, Colorado State University, Fort Collins, CO 80523 USA; 106 Institute of Genomics for Crop Abiotic Stress Tolerance, Department of Plant and Soil Sciences, Texas Tech University, Lubbock, TX 79409 USA; 107 GFZ Helmholtz Centre for Geosciences, Geomicrobiology Section, Telegrafenberg, 14473 Potsdam, Germany; 108 State Key Laboratory of Mycology, Institute of Microbiology, Chinese Academy of Sciences, Beijing, 100101 China; 109 Department of Ecology and Environmental Science, Umeå University, Umeå, Sweden; 110 State Key Laboratory of Vegetation and Environmental Change, Institute of Botany, Chinese Academy of Sciences, Beijing, China; 111 Key Laboratory of Vegetation Ecology, Ministry of Education/Jilin Songnen, Grassland Ecosystem National Observation and Research Station, Northeast Normal University, Changchun, 130024 China; 112 Heilongjiang Xingkai Lake Wetland Ecosystem National Observation and Research Station & Key Laboratory of Wetland Ecology and Environment, Northeast Institute of Geography and Agroecology, Chinese Academy of Sciences, Changchun, 130102 China; 113 Key Laboratory of Grassland Resources (Inner Mongolia Agricultural University), Ministry of Education, Hohhot, 010021 China; 114 School for Environment and Sustainability, University of Michigan, Ann Arbor, MI USA; 115 School of the Earth, Ocean and Environment, University of South Carolina, Columbia, SC 29208 USA

**Keywords:** Microbiology techniques, Microbial communities, Microbial ecology

## Abstract

Soil microbes drive ecosystem function and play a critical role in how ecosystems respond to global change. Research surrounding soil microbial communities has rapidly increased in recent decades, and substantial data relating to phospholipid fatty acids (PLFAs) and potential enzyme activity have been collected and analysed. However, studies have mostly been restricted to local and regional scales, and their accuracy and usefulness are limited by the extent of accessible data. Here we aim to improve data availability by collating a global database of soil PLFA and potential enzyme activity measurements from 12,258 georeferenced samples located across all continents, 5.1% of which have not previously been published. The database contains data relating to 113 PLFAs and 26 enzyme activities, and includes metadata such as sampling date, sample depth, and soil pH, total carbon, and total nitrogen. This database will help researchers in conducting both global- and local-scale studies to better understand soil microbial biomass and function.

## Background & Summary

Soil microbes, particularly bacteria and fungi, are critical for biogeochemical cycling and Earth’s planetary health^1^. Our understanding of soil microbes has been developed primarily through local and regional studies that use methods such as DNA metabarcoding and metagenomics, phospholipid fatty acid (PLFA) extractions, and enzyme activity assays to study microbial community composition and their role in ecosystem processes^2–4^. Efforts to collate global soil DNA metabarcoding databases have vastly improved our understanding of belowground microbial diversity patterns and species’ distributions^5–8^. However, quantifying microbial biomass and function remains challenging based on DNA sequencing alone^9^. PLFA and enzyme activity assays have been performed by scientists for decades, providing, among other things, the ability to estimate carbon stored in microbial biomass and measure *in situ* microbial community functioning^2,10^. These methods complement emerging DNA technologies and are crucial for understanding the potential impact of global change factors on carbon storage and other critical biogeochemical processes^9–11^.

PLFA assays involve measuring fatty acids associated with phospholipids of cell membranes^3,9,12^. PLFA molecules differ in factors such as fatty acid chain length, degree of saturation, branching, and functional group modifications^11^. To a certain degree, different PLFAs can be used as biomarkers of taxonomic groups such as fungi, protozoa, Gram-negative bacteria, and Gram-positive bacteria^10,11,13,14^, making it possible to use changes in the fatty acid composition of PLFAs to estimate broad shifts in microbial communities. Phospholipids are degraded rapidly after cell death, allowing PLFA assays to target living organisms^15^. Additionally, conversion factors have been developed that allow PLFAs to be used to estimate microbial carbon content^10,16,17^. Although PLFAs cannot provide taxonomic resolution equivalent to other quantitative biomass estimation methods, such as quantitative PCR, PLFA-based biomass estimates can be more reliable in many situations^9,18^. Additionally, PLFA analysis is a relatively cost-effective way to measure soil microbial community biomass and composition^10^ and has been widely employed since the early 1990s^3,19^.

Soil enzyme activity assays measure the oxidative or hydrolytic catalysis of organic matter substrates by enzymes in soils, the majority of which are thought to be extracellular^20,21^ and largely released by microbes but also by plant roots. Extracellular soil enzymes degrade organic polymers to liberate bioavailable forms of nutrients required for metabolism and growth^22,23^. These assays aim to quantify the maximum potential enzyme activity by incubating soil samples in the lab and, most commonly, colourimetrically or fluorometrically measuring the release of a chromophore or fluorophore from the oxidation or hydrolysis of dye-conjugated substrates^2,22^. It is important to note that these assays measure the maximum potential activity rather than *in situ* activity, which is influenced by temperature, soil pH, substrate availability, and other soil and ecosystem properties^22,23^. Due to substrate specificity, different enzyme activities relate to the acquisition of different products categorizable by macromolecular type and nutrient element. For example, β-glucosidase degrades cellobioside, and phosphomonoesterases — often referred to by the broader enzyme class term of phosphatases — hydrolyse phosphate monoesters^24^. Similar to PLFA assays, soil enzyme assays have been performed for many decades^25^.

Despite the long history of PLFA and enzyme activity assays, data access has largely been restricted to relatively small local and regional scales. Larger-scale global research networks and analyses are becoming increasingly important for complementing local-scale research to better understand and tackle Earth’s global-scale environmental challenges^26^. Global-scale meta-analyses of microbial biomass carbon (MBC) have revealed that MBC shows complex biogeographical patterns and is highly sensitive to environmental change and human land use^27–30^. However, the underlying data used to estimate microbial biomass and function (i.e., the raw PLFA and enzyme measurements) are rarely available in meta-analyses, making it difficult to use these datasets to answer new and targeted research questions.

To improve the availability of soil microbial data, Smith, *et al*.^31^ released an open call for collaboration to develop a global database of soil PLFA and potential enzyme activity measurements. Many scientists with data collected from 89 countries answered this call and here we provide access to the developed database. We provide a full description of the database, along with analyses assessing the coverage of environmental space, remaining data gaps, and potential biases that users should be aware of. This database will allow researchers to investigate critical questions at both local and global scales to better understand patterns of microbial biomass and function. We also hope that data gaps revealed in this database will inspire further research in data-limited regions so that geographical biases can be reduced in the future.

## Methods

Following the open call for collaboration by Smith, *et al*.^31^, georeferenced data from PLFA and enzyme assays of soil samples were provided by interested collaborators. Additional PLFA data were sourced from the United States National Ecological Observatory Network^32^. We also added data from several sources that reported individual PLFA measurements^33–37^, as well as a recent study with a large enzyme dataset^38^. Where necessary, data were extracted from figures using DataThief ^39^. We did not perform an additional exhaustive formal literature search because very few studies have reported measurements of individual PLFA biomarkers. Only samples with geographical coordinates were included. Data from experimental plots were excluded, as well as those from samples solely consisting of leaf litter.

Authors performed PLFA and enzyme activity assays using numerous well-recognised methods, with the cited methods used by each study listed in the database. Full sample collection and processing methods can be found in the original publications for previously-published samples (DOIs provided in the database). Methods for unpublished samples are included in this publication as Supplementary Information. The majority of PLFA assays were performed using variants of Bligh and Dyer^12^ lipid extraction methods and gas chromatography-mass spectrometry, following Frostegård, *et al*.^19^. Several contributed datasets used ester-linked fatty acid measurements, following Schutter and Dick^40^. Although these two methods recover comparable compositional signals, ester-linked fatty acid measurements have concentrations approximately twice as high^41^. Therefore, we divided values from these samples in half to scale them appropriately. Enzyme activities were assayed using colourimetric and/or fluorometric methods^22^, and in the case of urease, with the natural substrate (i.e., urea). Assays were assumed to be performed under optimal conditions of substrate according to best practices, and varied in assay incubation temperatures from a standardised temperatures or a temperature that reflected *in situ* conditions (e.g., mean annual temperature).

We thoroughly cleaned and standardised the database by first converting all PLFA units to nmol g^−1^ soil and enzyme activity units to nmol h^−1^ g^−1^ soil. We also checked all other variables and converted variable categories and units where needed. Sample depths listed as O horizon, A horizon, or “organic” were classed as 0–10 cm. Then, where possible, missing important data (e.g., enzyme reaction temperature) was obtained by re-contacting data contributors or examining publications. We examined the range of values in all variables to look for errors and outliers. A small number of samples contained negative enzyme activity values, which we replaced with zero, and percentages greater than 100, which we capped at 100. It was not possible to evaluate the precision or accuracy of provided sample coordinates. However, we used the “coordinateCleaner” R package v3.0.1^42^ to ensure all coordinates correspond to the correct coordinate reference system (WGS84), and to flag any potential errors such as those with equal absolute latitude and longitude or those within a 100 m radius of country centroids or capitals. All included samples passed coordinate validity checks.

We included all PLFAs and enzymes in the final database for which, once the data sources were merged, there were at least 100 data points available. We extracted the continent, country, and biome^43^ information for each sample location using the “terra” (v1.7–78), “sf” (v1.0-19), and “rnaturalearth” (v1.0.1) packages in R version 4.4.1^44–47^.

## Data Records

The database is available as a .xlsx file on Figshare^48^. The file includes tabs with the names of all PLFAs and enzymes (including Enzyme Commission numbers) for which data are available, and the number of samples available for each. The metadata, PLFA data, and enzyme data for each sample are provided in separate tabs which can be linked by the “sampleID” column. All column names are programming-language friendly.

**Fig. 1:**
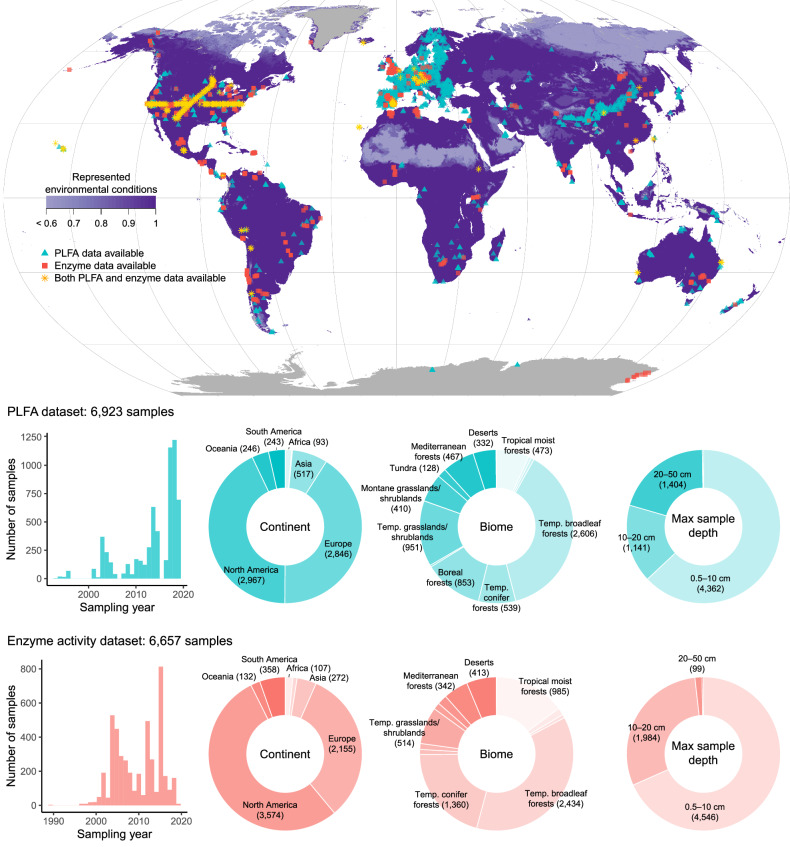
The PLFA and enzyme activity database contains 12,258 samples from 3,743 locations. The purple colour gradient shows the degree to which climatic space is represented by the samples (scale of 0 to 1, see Technical Validation section). Grey regions do not have sufficient climate data available to evaluate climatic representation. The histogram and donut plots show number of samples (in parentheses) from the PLFA (blue) and enzyme (red) datasets collected in different years, from different continents, from different biomes^43^, and within different categories of maximum sample depth. Only large segments of donut plots are labelled. Temp. = temperate.

In total, there are data for 12,258 soil samples from 3,743 unique locations (Fig. 1). There are 6,923 samples with PLFA data (for 113 PLFAs), 6,657 samples with enzyme activity data (for 26 enzyme groups), with 1,322 samples containing both. Data from 627 samples (5.1%) have not previously been published. Samples were collected between 1989 and 2019 (Fig. 1). Of all samples, 10,436 (85%) are from natural ecosystems, and 1,822 (15%) are from managed (e.g., agriculture, plantation, urban) ecosystems. Data are predominantly from North America and Europe, but samples are available from all continents, including 70 samples from Antarctica (Fig. 1). Temperate and boreal biomes are the best represented, but many samples are also available from tropical moist broadleaf forests, particularly in the enzyme dataset. Montane grasslands, Mediterranean forests and deserts are also reasonably well represented. Most samples were collected with a maximum sampling depth between 0.5 and 20 cm, but the PLFA dataset also contains high representation of data from up to 50 cm deep (Fig. 1).

Fourteen of the most commonly assessed PLFAs and five of the most commonly measured enzymes are very well represented in the database, with between ~2,600 and ~6,800 samples available for each (Table 1). Data are also available for an additional 99 PLFAs and 21 enzyme categories. Metadata relating to sample depth are available for 100% of PLFA samples and 99.9% of enzyme samples, and data relating to sampling year, sampling month, and soil pH are available for 47–90% of PLFA samples and 60–86% of enzyme samples (Table 1). Metadata of other soil properties (carbon, nitrogen, moisture and bulk density) and elevation are also available for many samples (Table 1).

**Table 1:** Data available for 14 of the most commonly assessed PLFAs and five of the most commonly measured enzymes.

Name	Putative group	# samples	Metadata availability (%)								
			Depth	Year	Month	pH	C	N	Moisture	BD	Elev.
**PLFAs**											
c18:2ω6c	Fungi	6,612	100.0	89.2	85.5	46.5	4.0	29.8	2.4	1.3	32.8
cy17:0	GN bacteria	6,523	100.0	89.4	85.6	44.9	4.1	29.6	3.7	2.3	33.5
cy19:0		6,612	100.0	89.2	86.2	45.4	4.0	30.2	3.6	2.3	33.2
c16:1ω7c		5,600	100.0	88.7	87.8	45.9	4.7	31.8	2.9	2.7	36.1
c18:1ω7c		4,829	100.0	88.1	83.1	42.9	0.0	24.1	2.3	1.7	43.5
a15:0	GP bacteria	6,764	100.0	89.5	85.9	45.7	3.9	30.8	3.5	2.2	31.1
a17:0		4,962	100.0	89.6	84.7	44.4	4.3	27.4	0.6	3.1	43.7
i14:0		3,902	100.0	94.9	93.8	37.8	4.2	21.5	0.0	1.8	51.2
i15:0		6,883	100.0	89.7	86.1	47.0	3.9	32.0	3.5	2.2	32.5
i16:0		6,860	100.0	90.5	86.9	47.2	3.9	32.6	3.5	2.2	32.6
i17:0		6,665	100.0	89.6	85.9	45.3	4.0	30.7	3.6	2.3	31.4
c16:0–10Me	GP bacteria (act.)	5,401	100.0	97.8	93.4	46.1	3.1	31.2	0.6	1.9	41.4
c17:0–10Me		4,394	100.0	98.2	92.7	47.5	4.9	28.5	0.0	1.6	50.2
c18:0–10Me		5,694	100.0	87.6	83.4	39.1	2.9	24.8	0.6	1.8	39.3
All available PLFAs		6,923	100.0	89.7	86.2	46.8	3.8	32.3	3.5	2.2	32.3
**Enzymes**											
β-glucosidase	C acquisition	6,487	99.9	66.5	59.2	86.4	9.3	51.9	16.3	6.1	22.4
Cellobiohydrolase		2,638	100.0	75.6	75.3	89.2	0.0	56.4	23.2	11.2	4.1
Leucine aminopeptidase	N acquisition	3,278	99.8	76.9	62.4	92.6	17.4	59.8	30.5	6.3	20.9
N-acetylglucosaminidase		5,110	99.8	59.2	49.8	84.4	11.1	42.3	20.8	5.8	18.4
Acid phosphatase	P acquisition	4,445	100.0	85.3	74.7	90.8	12.4	67.4	23.9	8.9	12.2
All available enzymes		6,657	99.9	67.3	60.1	86.3	9.1	52.3	16.0	6.0	22.9

Microbial groups and nutrient acquisition type classifications are putative only and specific to soil samples, based on information from Willers, *et al*.^11^, Joergensen^57^ and Dick^24^. GN = Gram-negative, GP = Gram-positive, act. differentiates Gram-positive bacteria that are actinobacteria. The number of samples available for each PLFA/enzyme and all available PLFAs/enzymes in the database is shown alongside the percentage of those samples for which metadata are available. C and N refer to soil carbon and nitrogen, moisture = gravimetric soil moisture content, BD = soil bulk density, and elev. = elevation.

## Technical Validation

We checked the database for erroneous outliers by calculating the interquartile range (IQR) of values within each biome for each PLFA/enzyme, and flagging values greater than 5 times the IQR. PLFA and enzyme data are often left-skewed, with our database being no exception, and many samples contained flagged values. We scanned the flagged values to look for patterns regarding the assay methods used or the study for which the samples were collected. No patterns were evident, and so all data were retained.

We assessed climatic space represented by the samples by assessing the degree of extrapolation in multidimensional space, following methods described in van den Hoogen, *et al*.^49^. Briefly, we extracted values for 19 bioclimatic layers from CHELSA^50^ for each point location in the dataset, then transformed all values into principal component (PC) space. Next, we assessed whether each pixel value of the global bioclimatic layers fell within or outside convex hulls for each of the bivariate combinations from the first five principal components. These five PC axes collectively covered more than 90% of the sample space variation. We plotted the proportion of times that each pixel fell within the convex hulls on a map to evaluate the degree to which climate space is represented in the database (Fig. 1). As with many other large ecological datasets^51^, global coverage of the data remains geographically and climatically uneven. In particular, substantial portions of Africa, South America, Asia, Antarctica, and ecosystems at high northern latitudes are currently under-sampled (Fig. 1).

**Fig. 2:**
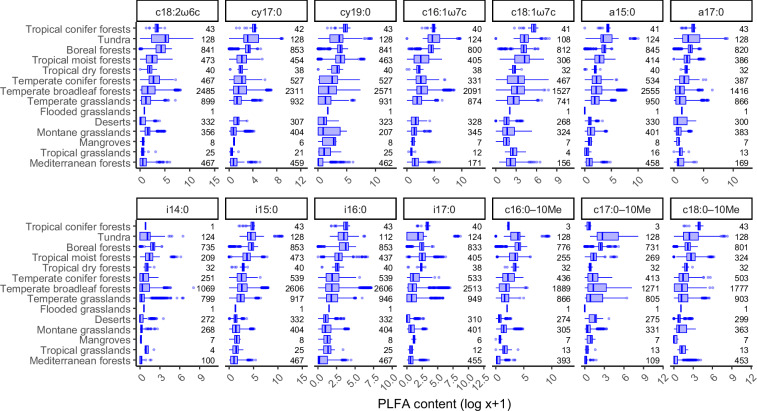
Variation in PLFA content across biomes^43^. Boxes show median and interquartile range, with whiskers 1.5 times the interquartile range. Biomes are ordered according to the median value across all 14 PLFAs. Numbers show the sample size in each category.

**Fig. 3:**
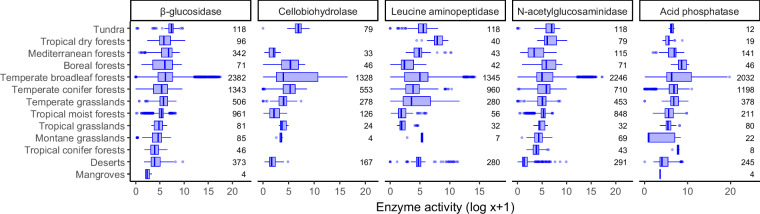
Variation in soil enzyme activity across biomes^43^. Boxes show median and interquartile range, with whiskers 1.5 times the interquartile range. Biomes are ordered according to the median value across all five enzyme activities. Numbers show the sample size in each category.

PLFA contents and enzyme activities vary across biomes (Figs. 2, 3). Overall, PLFA values are relatively high in tropical conifer forests, tundra, and boreal forests (Fig. 2). Similarly, soil enzyme activities are high in tundra and boreal forest samples, but some enzyme activities are also high in soils from tropical dry forests and Mediterranean forests (Fig. 3).

**Fig. 4:**
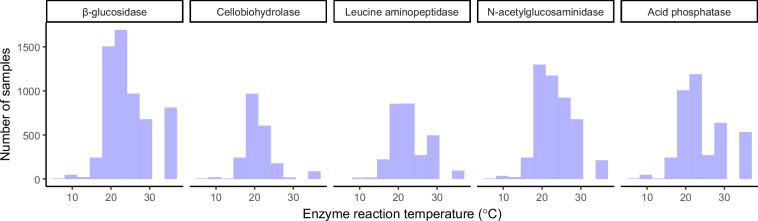
The distribution of temperatures used to assay enzyme activity.

Soil enzyme activities were measured using a variety of incubation temperatures ranging between 8 °C and 37 °C (Fig. 4). Assay incubation temperature data are available for 91% of samples (6,025). More details on the variation in assay temperature and its potential influence on activity rates are provided in the Usage Notes section.

## Usage Notes

All samples are georeferenced, and so data at the pixel level relating to climate^50,52^, soil properties^53^, and land cover^54^, for example, could be extracted from publicly-available global geospatial layers. Many samples contain field-collected metadata (Table 1), but for those that do not, available geospatial layers could also be used to fill in gaps. However, users should be aware that these geospatial layers are predictions associated with various uncertainties^55^.

**Fig. 5:**
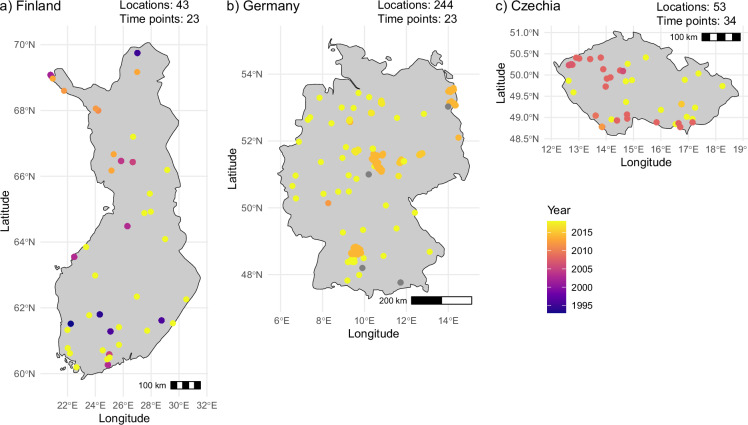
Examples of countries that have data available for multiple years. Points are coloured according to the sampling year; points are grey where sampling year is unavailable. Text indicates the number of locations in each country, and the number of unique time points (year–month combinations) that samples were collected at.

Because the database includes data collected over 30 years (1989 to 2019), there is potential to conduct time-series analyses. For example, some densely sampled countries contain samples spanning 6 to 25 years (Fig. 5). Additionally, 298 of the unique sampling locations (8%) contain data from more than one time point (unique month-year combinations), with the highest number of time points for a single location being 17. Time-series analyses are becoming increasingly important to track the response of organisms to global change factors, and such datasets are highly valuable^56^.

One important property of PLFA content measurements is that they are additive. As mentioned earlier, different PLFAs can be associated with different microbial functional groups, such as fungi, Gram-negative bacteria, actinobacteria, and other Gram-positive bacteria^11,13,57^. PLFA measurements that are markers of the same group can therefore be summed to estimate biomass of those groups and the ratios of different groups examined. However, the accuracy of some commonly used classifications has been questioned^10,11^. For example, it is recognised that the PLFA 16:1ω5, sometimes used as a marker of arbuscular mycorrhizal fungi, also occurs in significant amounts in bacteria^58,59^. Also, some PLFAs can be good indicators of certain groups in some (agro)ecosystems, but not others^10^. For example, 18:1ω9 can be a good indicator of fungi in soils under forest but not agricultural land use^10^. It is important that data users review the latest literature and be aware of any potential errors with classifications.

The magnitude of enzyme activities measured in soils can be strongly influenced by several methodological parameters, in particular assay temperature, pH, and substrate concentration^60–64^. It is important that the impact of different assay conditions on enzyme activity is considered when analysing and interpreting these data, because whilst there are recommendations for best practices in soil enzyme activity assays^65^ these are not always strictly adhered to^62^. We made the assumption that soil enzyme activities were assayed at non-rate limiting substrate concentrations (i.e., activities approximate maximum catalysis rate (V_max_)), which is recommended^22,66^ to ensure that the activity assayed is independent of substrate-concentration^67^. As a result, soil enzyme activities assayed at non-rate liming activities reflect inherent differences in activity (V_max_) of a soil sample^62^. Confirming that substrate concentrations approximate V_max_ requires soil sample-specific substrate saturation curves to be calculated^62,68^. We assume in good faith that individual labs have confirmed that the substrate concentrations used achieve V_max_, which can vary by soil as well as assay conditions such as buffer and temperature^22,68,69^.

Similarly, as enzyme activities are standardised by maximizing activity, we assume that individual researchers confirmed for their soils that the use of assay pH^70^, substrate concentration^22,62^ and matrix (e.g., buffer)^68^ ensure maximization of assayed activity. Though there are multiple issues with assumptions made in assay conditions that maximize activities^68,70^, such assumptions are ubiquitous in soil enzyme activity assays. Thus, this is a potential issue that impacts all soil enzyme activity data and not just our dataset. Our dataset reflects the best possible quality to-date in the field, even though there are methodological improvements needed^71^.

Though most researchers standardise enzyme activity assay by using assumed assay conditions that maximise activities to measure the maximum potential activity^23,72^, others perform assays under temperature and pH conditions that match those at the locations at which the samples were collected, in an attempt to better assess *in situ* activities^22,23^. For some research questions, this may be the more appropriate approach^71^. The methods used to assay soil enzyme activities in our database include a mix of both approaches, which reflects the reality of methodology diversity in soil enzyme activity assays used by researchers globally. To ensure transparency and enable interpretation of enzyme activities based on assay conditions, we have provided all the metadata available for each sample (e.g., assay temperature and pH) as well as soil properties so that database users can incorporate these variables into models in a way that is most appropriate for the analyses being performed. For example, it is possible to use temperature sensitivity models (e.g., Arrhenius equations) to normalize activity for different enzymes based on known enzyme kinetics^60^. To date, no well-established standardisation methods currently exist that take into account the full complexity of the assay parameters of temperature, matrix type and substrate concentration that may impact absolute values of assayed enzyme activities^62,64,73^. This is a clear need for soil enzymology.

Finally, users should be aware of the biases in the database towards certain geographical regions and climatic zones. The impact of both geographical and climatic biases on model outputs should be carefully explored when conducting any analysis^74^. Data thinning or other bias correction approaches may be required. Additionally, many PLFAs and enzyme activities contain measurements from multiple samples collected at the same location, and so users must decide on the most appropriate way to treat these values.

## Supplementary information

Supplementary methods

## Data Availability

Code used to conduct technical validation analyses and create the figures is available on Figshare^48^.
