# Supplementary material for: A global database of soil microbial phospholipid fatty acids and enzyme activities
Source: Sci Data. 2025 Sep 26;12:1568. doi: 10.1038/s41597-025-05759-2 (PMC12475190; doi:10.1038/s41597-025-05759-2)
Supplement: Supplementary file 1 — Supplementary methods [file 41597_2025_5759_MOESM1_ESM.pdf]

Methods for unpublished samples

Contents

Samples 186–254 1
Samples 1716–1727 2
Samples 1728–1747 and 3612–3619 2
Samples 3194–3197 3
Samples 3249–3278 3
Samples 3429–3433, and 10541–10617 4
Samples 3492–3611 4
Samples 3652–3701 5
Samples 4461–4472 5
Samples 4541–4621 6
Samples 4622–4729 6
Samples 6762–6773 and 6908 7
Samples 7030–7036 and 12175–12178 8
Samples 10618–10662 8
Samples 10663–10668 9
Samples 11361–11364 10
References 11

Samples 186–254

These samples were collected from a study conducted in 23 radial plots (10 m radius each) within the Ranch Brook watershed in Stowe, Vermont. Ranch Brook is a 9.6 km^2 basin on the southeastern slope of Mount Mansfield, with elevation ranging from 335 to 1173 meters above sea level (m asl). The forest composition shifts from northern hardwoods to spruce-fir around 750 m asl. At lower elevations, dominant overstory species include American beech (*Fagus grandifolia* Ehrh.), sugar maple (*Acer saccharum* Marsh.), red maple (*Acer rubrum* L.), and yellow birch (*Betula alleghaniensis* Britt.). Higher elevations are characterized by red spruce (*Picea rubens* Sarg.) and balsam fir (*Abies balsamea* (L.) Mill.).

We distributed the 23 plots across north-, east-, and south-facing aspects to capture a range of elevations and forest types. Within each plot, we randomly selected three locations and collected soil samples from the top 10 cm. All samples were collected over a three-day period in early July under dry conditions. In the field, we visually assessed horizon depths within the top 10 cm, kept samples cool during transport, and sieved them to 4 mm due to high organic matter content. We manually removed remaining roots and homogenized the soil before subsampling 2 g for enzyme assays. Subsamples were stored at –18°C until analysis.

To evaluate potential microbial activity and function, we assayed seven ecologically relevant extracellular enzymes: β-glucosidase, α-glucosidase, cellobiohydrolase, β-xylosidase, β-N-acetylglucosaminidase, acid phosphatase, and peroxidase. We used 4-methylumbelliferyl (MUB)-linked substrates for the first six

enzymes—β-D-glucopyranoside, α-D-glucopyranoside, β-D-cellobioside, 7-β-D-xylopyranoside, N-acetyl-β-D-glucosaminide, and phosphate disodium salt—as described by Saiya-Cork, et al. [1]. We used 0.30% hydrogen peroxide to assess peroxidase activity. We performed six fluorometric enzyme assays, reading fluorescence at 365 nm excitation and 450 nm emission using a BioTek SynergyHT microplate reader, 60 seconds after adding 20 μL of 0.5 M NaOH to stop the reaction. We measured peroxidase activity colorimetrically at 460 nm. We calculated enzyme activities as μmol product per gram dry soil per hour (μmol g^{-1} h^{-1}).

Samples 1716–1727

All soil samples were collected from four replicate alpine meadow plots on the Qinghai-Tibetan Plateau in Golog, Qinghai Province, China, in August 2018, over a two-week period. Each plot measured 10 × 10 m and was at least 100 m apart between plots. Within each plot, three random soil subsamples were collected using a 50 mm diameter soil auger to a depth of 10 cm. To prevent cross-contamination, the auger was cleaned with 75% ethanol between each collection. All soil subsamples were stored at 4°C between collection until return to the laboratory.

Upon return to the laboratory, all soil subsamples were separately sieved through a 2 mm mesh sieve and stored at 4°C. The sieve was sterilised with 75% ethanol between subsamples to prevent cross-contamination. A portion of sieved soil from each subsample was taken for the analyses of: pH (1:2 soil to water ratio solution), total organic carbon and nitrogen using an elemental analyser, while soil β-glucosidase and phosphomonoesterase activities were measured using a spectrophotometric microplate reader according to the methods as described by Jackson, et al. [2] within three weeks of soil collection. The remaining soil was freeze-dried for subsequent phospholipid fatty acid (PLFA) analysis using a gas chromatograph according to the methods of Bligh and Dyer [3] as described by Frostegård and Bååth [4].

Samples 1728–1747 and 3612–3619

Sampling was carried out in natural habitats, with no visible anthropogenic impact. At each sampling location, 20-40 individual soil samples were collected from a depth of up to 10 cm and approximately 1000-2500 m^2 size area, depending on habitat size and heterogeneity, and pooled. Five grams of soil were subsampled for fatty acid analyses and dried within 12 hours.

Lipids were extracted from 5 g of dried soil in a one-phase solution of chloroform, methanol and citrate buffer [5]. The supernatant was split into two phases by the addition of chloroform and citrate buffer. 2.5 ml of the lipid containing phase was collected and dried under a flow of nitrogen gas at 37°C. The lipids were then fractionated on pre-packed silica columns (Thermo Fisher HyperSep™ silica SPE columns, 500 mg), into neutral, intermediate and polar lipids (the latter containing the phospholipids) by elution with chloroform, acetone and methanol, respectively. Phospholipids and neutral lipids were dried and an internal standard (nonadecanoic acid methyl ester, Me19:0) was added. The lipids were dissolved in 1 ml toluene:methanol (1:1 v/v) and then subjected to mild alkaline methanolysis by adding 1 ml 0.2 M potassium hydroxide and incubated at 37°C for 15 min. When the samples had cooled to room temperature, 2 ml hexane:chloroform (4:1 v/v), 0.3 ml 1 M acetic acid and 2 ml distilled water were added, followed by 5 min centrifugation at 3000 g. The lipid phase was dried under a flow of nitrogen gas at room temperature and then dissolved in hexane before analysis using gas chromatography and flame ionization detection (FID), as described by Frostegård, et al. [6]. Fatty acid content was then calculated on the basis of the internal standard.

Samples 3194–3197

Soil sampling was carried out in December 2016 (at the end of the wet season) in a natural area mainly covered by *Triodia epactia, Indigofera monophylla, Eucalyptus gamophylla* and *Corymbia hamersleyana* in the Pilbara region, Northwestern Australia. Soils were taken from 2x2 m plots that were at least 20 m apart from each other. From each plot, a bulked soil sample composited from three individual samples was collected from the top 5 cm of the soil surface.

Samples were air-dried, sieved (2 mm mesh), and divided in two subsamples. One was used for physical and chemical analysis, and the other was stored at 4°C for 2 weeks and sent to Microbiology Laboratories Australia (https://microbelabs.com.au) for PLFA analysis. All analyses followed the methods described in Muñoz-Rojas, et al. [7].

Specifically, PLFA analysis followed the procedure of Buyer, et al. [8]. Briefly, fatty acids were extracted from soil subsamples of 5 g with a modified Bligh-Dyer extraction using 19 ml of a 1:2:0.8 mixture of chlororm:methanol:phosphate buffer extractant. PLFAs were then separated by solid phase extraction columns (0.5 g of Si) (Supelco Inc., Bellefonte, Pa.). The resulting polar lipid fraction was then subjected to mild alkaline methanolysis, and samples were analysed using a Hewlett Packard 6890 gas chromatograph with a 25 m Ultra 2: (5%-phenyl)-methylpolysiloxane column (J&W Scientific, Folsom, CA). Peaks were identified using bacterial FA standards and MIDI peak identification software (MIS; Microbial ID Inc.). PLFAs were quantified relative to nonadecanoic acid (C19:0) as internal standard by comparison of peak areas with those of the 19:0 peak.

Samples 3249–3278

This study was conducted at the Drought and Root Herbivore Impacts on Grassland (DRI-Grass) experimental facility. The DRI-Grass experiment started in June 2013 and is located in a seminatural mesic grassland. The grassland is dominated by native and exotic C_4 species (e.g., *Cymbopogon refractus* (R.Br.) Camus, *Cynodon dactylon* (L.) Pers., *Eragrostis brownii* (Kunth) Nees, and *Paspalum notatum* Flüggé). The remaining vegetation is a mixture of native and exotic C_3 grasses (e.g., *Lolium perenne* L. and *Microlaena stipoides* (Labill.) R.Br.) and forbs (e.g., *Hypochaeris radicata* L., *Plantago lanceolata* L.). The experiment consists of 48 plots (1.9 × 2.5 m). In this study, we used a subset of 6 control plots (i.e., no precipitation or root herbivore treatment implemented).

Soil samples (0–10 cm depth) were collected five times between April 2014 and April 2016 (April 2014, January 2015, March 2015, September 2015, and April 2016), using a soil corer (1 cm diameter). Eight soil subsamples were randomly collected in each plot and bulked. Samples were then preserved at 5°C (microbial enzyme activity and biomass) before subsequent analyses within 2 weeks.

Assays were conducted by homogenizing 1 g of soil in 30 mL of 50 mmol/L sodium acetate buffer at pH 6.5 for 1 minute. The homogenized solutions were then added to 2-mL 96-deep-well microplates. Control replicates of soil slurry and 4-methylumbelliferone (MUB) or 7-amino-4-methylcoumarin (MUC) standard curves of 0–100 µM were included in each sample. Soil slurries with fluorometric substrates (Sigma-Aldrich, St. Louis, Missouri, USA; M9766 for AG, M3633 for BG, M7008 for XYL, M6018 for CBH, M2133 for NAG, L2145 for LAP, M8883 for PHOS, and M7133 for AS) were then incubated for 1.5 h at 35°C. Following incubation, the supernatant solution was transferred into corresponding wells in a black, flat-bottomed 96-well plate. The plates were then scanned on a microplate fluorometer (2300, EnSpire Multilabel Reader, PerkinElmer, Boston, Massachusetts, USA) using an excitation wavelength of 365 nm and an emission wavelength of 450 nm.

Samples 3429–3433, and 10541–10617

Samples 3429–3433 are part from the Nutrient Network [9] with the sublocation at Bad Lauchstädt (51°23'32.4"N 11°52'40.6"E). The plot layout of the Nutrient Network consists of 5 × 5 m plots arranged in a randomized block design, typically including treatments for nutrient addition (e.g., nitrogen, phosphorus, and potassium in all combinations) and control plots. In this case, no herbivory treatment (no fences) was included. Per plot, three subsamples were collected using a 5-cm diameter corer to a depth of 12 cm [10]. Afterwards, soil samples were sieved at 2 mm and stored at −20°C until analysis.

Samples 10541–10617 are part of the project Biodesert, funded by the European Research Council [ERC grant agreement 647038]. This subgroup of 77 samples come from drylands of 24 countries with different levels of grazing (ungrazed, low and medium grazing). Soil collection was carried out with a stratified random procedure, where ten quadrats (50 x 50 cm) were randomly placed at each plot, under vegetation and in open areas. Five soil samples were collected from each quadrat with 145 cm^3 soil cores (0-7.5 cm depth) that were homogenized in the field to reach a composite topsoil sample, which was sieved (2 mm mesh) and frozen at −20°C. A detailed description of the sampling campaign can be found in Maestre, et al. [11].

Phospholipid fatty acid (PLFA) analyses were conducted following the protocol from Frostegård, et al. [12], with modifications according to Bligh and Dyer [3]. Approximately 5 g of fresh soil was extracted using a one-phase mixture of chloroform, methanol, and citrate buffer (1:2:0.8, v/v/v). Lipids were separated by solid-phase extraction (silica columns) into neutral lipids, glycolipids, and phospholipids. The phospholipid fraction was transesterified to produce fatty acid methyl esters (FAMEs), which were analyzed using gas chromatography with flame ionization detection (GC-FID; Clarus 680, PerkinElmer) equipped with a Supelco SP-2560 capillary column (100 m length, 0.25 mm diameter, and 0.25 µm film thickness).

Samples 3492–3611

Samples 3492–3611 originate from a field experiment conducted at the Tachenhausen Experimental Farm near Stuttgart, Germany (48.649800 N, 9.387500 E, 330 m a.s.l.).

The soil is a Stagnic Cambisol[13] with a very fine sandy loam texture. The field has an average $pH_{(H_2O)}$ of 6.5 and a soil organic carbon content of 14 g kg^{-1} soil. In the field trial, the effects of tillage (no-till/NT vs reduced tillage/RT) and soil coverage (cover crops/CC vs bare fallow/noCC) on soil properties were compared in a full factorial design with three complete blocks of plots (strips of 6 m by 100 m) arranged in a split plot design (total of 12 plots).

Soil samples were taken in February (3492–3515), April (3516-3539), July (3540–3563), October (3564–3587) 2015 and March 2016 (3588–3611) in 0–5 and 5–20 cm depths with an auger, from around eight locations inside each of the twelve plots and pooled per plot and depth. The samples were sieved at 5 mm and stored at −20°C until analysis. Of this dataset, samples from February (3492–3515) and October (3564–3587) are published in Hallama, et al. [14], while the samples of the other timepoints 3516–3563 and 3588–3611 were not published until now.

Potential activities of acid phosphomonoesterase (EC 3.1.3.1), phosphodiesterase (EC 3.1.4.1), β-D-glucosidase (EC 3.2.1.21) and N-acetyl-glucosaminidase (EC 3.2.1.52) were determined using fluorescent 4-methylumbelliferone substrates based on Marx, et al. [15], modified by Poll, et al. [16]. The substrates were obtained from Sigma–Aldrich, St. Louis, USA, except for the phosphodiesterase substrate, which was obtained from Carbosynth, Compton, UK.

For the analysis, 1 g of soil was ultra-sonicated at 50 J s^{-1} for 120 s in 50 ml of autoclaved H_2O. Fifty µl of soil suspension, 50 µl MES buffer (0.1 M MES-buffer, pH 6.1) and 100 µl substrate were pipetted onto microplates and incubated at 30°C. The increase in fluorescence over time (slope) was measured at 5 intervals over 180 min at 360/460 nm on a Microplate Fluorescence Reader (FLX 800, Bio-Tek Instruments, USA) and converted into nmol substrate g $soil^{-1}$ h^{-1} using a sample-specific standard curve with 4-methylumbelliferone added to the soil suspension.

Phospholipid fatty acids (PLFA) and neutral fatty acids (NLFA) (Frostegård, et al. [17], modified according to Kramer, et al. [18]). Fatty acids were extracted from 2 g soil [19], based on the method of Bligh and Dyer [3] and modified by White, et al. [20]. Fatty acid methyl-esters were stored at −20°C until identification by chromatographic retention time and comparison with a standard mixture of qualitatively defined fatty acid methyl-esters ranging from C11 to C20 (Sigma Aldrich, Germany).

Samples 3652–3701

Soil samples 3652–3687 were collected in October 2016 from 50 × 50 m plots established at each study site in lowland rainforest and riparian lowland rainforest within the Harapan region, Jambi Province, southwest Sumatra, Indonesia [21].

Soil samples 3688–3701 were collected in May 2009 in southern Ecuador, at the northern boundary of Podocarpus National Park, located on the eastern slopes of the Andes. Three study sites were selected along an altitudinal gradient at elevations of 1000 m, 2000 m, and 3000 m above sea level, representing old-growth premontane rainforest, old-growth lower montane rainforest, and upper montane rainforest, respectively [22].

Environmental variables and phospholipid fatty acids (PLFAs) were analysed for all samples. For each plot, composite samples were prepared from five (IDs 3652–3687) to six (IDs 3688–3701) soil cores (Ah horizon), taken with a 5 cm diameter corer to a depth of 5 cm. Coarse roots and large organic debris, including seeds and twigs, were manually removed. Samples were kept on ice in the field and stored at −20°C until further processing.

Soil pH ($CaCl_2$) for all samples was measured using a digital pH meter. For samples 3652–3687, subsamples were dried at 65°C for 72 hours, milled, and analysed for total carbon (C) and nitrogen (N) concentrations using an elemental analyser (Carlo Erba, Milan, Italy).

PLFA extraction for all samples followed the protocol of Frostegård, et al. [17] using 4 g of soil. Lipids were fractionated on silica acid columns (0.5 g silicic acid, 3 mL). Fatty acid methyl esters (FAMEs) were identified based on chromatographic retention times compared with a standard mixture containing 37 FAMEs ranging from C11 to C24. Gas chromatographic analysis was performed on an AutoSystem XL equipped with a HP-5 capillary column (50 m × 0.2 mm inner diameter, film thickness 0.33 µm). The temperature program began at 70°C (held for 2 minutes), increased at 30°C/min to 160°C, then at 3°C/min to 280°C, where it was held for 15 minutes. The injector temperature was set to 260°C, and helium was used as the carrier gas. Individual PLFAs were expressed as a percentage of total PLFAs (relative nmol g^{-1} dry soil weight).

Samples 4461–4472

Three natural forests were investigated for soil health indicator monitoring. Two sites were sampled during August 2016 from Himalayan cold desert districts (Kaltse and Thiksey) and one from Avalahalli subtropical forest during March 2018 near Bangalore, India. In each site, four equal plots (replicates) of 100 m^2 (10 m x

10 m) were established with minimum 100 m distance from each other. Soil samples were taken in March 2016 from each plot. Samples were collected from ten sampling points along a z shaped trajectory at 0–10 cm depth and pooled to obtain four representative samples per site. Soil samples were sieved (< 2 mm) to remove visible roots and litter material and stored at 4°C until analysis. Dehydrogenase and urease activities were determined in duplicate soil samples collected from each treatment plot. Briefly, dehydrogenase activity was quantified by determining the concentration of triphenyl formazan (TPF) produced after incubation of soil samples (1 g each) with triphenyltetrazolium chloride for 24 h at 30°C. The intensity of the red colour was measured at 485 nm. Dehydrogenase activity was expressed as µg of TPF formed per gram of sample per 24 h [23]. Urease activity was determined as the amount of ammonia released after incubation of soil samples (5 g each) with urea for 2 h at 37°C [24].

Samples 4541–4621

Three areas were selected, each where land use changes occurred—from native forest to pasture and to sugarcane cultivation. The first area is located in the municipality of Jataí-GO (17º56′16″ S; 51º38′31″ W), the second in Valparaíso-SP (21º14′48″ S; 50º47′04″ W), and the third in Ipaussu-SP (23º05′08″ S; 49º37′52″ W). The selected areas represented a north-south transect through the South-Central region of Brazil.

Soil sampling at each of the three study sites was conducted in January 2013 using a sampling scheme based on the model proposed by Huising, et al. [25], adapted from Swift and Bignell [26]. This scheme involves collecting nine sets of samples (replicates) arranged in a grid pattern, with points spaced 50 meters apart for each land use type. Each sampling point for each land use at each location consisted of nine sub-samples taken with an auger from the 0–10 cm soil layer, at 3 m and 6 m from the central point. These nine sub-samples from each sampling point were mixed and homogenized, forming a composite sample for each sampling point. Thus, nine composite samples were obtained for each land use at the three study sites. A total of 81 composite field samples were placed in ventilated plastic bags and sent to the laboratory on the same day. Part of these same samples was used for enzymatic analyses and stored in coolers with ice for transport, then kept refrigerated at 4°C.

The activity of acid and alkaline phosphatases in the samples was determined using the colorimetric method according to Tabatabai [27], which aims to mimic the environmental conditions of the sample so that the enzyme expresses its potential under natural conditions in the laboratory. The samples were incubated in a water bath for one hour in a buffered solution of p-nitrophenyl phosphate and toluene at 37°C, and measured by spectrophotometry at 400 nm. The concentration of p-nitrophenol released after the enzymatic reaction was estimated using a standard curve with levels of 0, 10, 20, 30, 40, 50, 60, 80, and 100 µg p-nitrophenol. The results were expressed in µg p-nitrophenol g dry soil h^{-1}.

To analyze β-glucosidase activity, also according to Tabatabai [27], the samples were incubated for one hour at 37°C in a buffered solution of p-nitrophenyl β-D-glucoside and toluene. The reading was taken by measuring the intensity of the yellow colour using a spectrophotometer at 400 nm. The concentration of p-nitrophenol released after the enzymatic reaction was estimated using a standard curve with levels of 0, 10, 20, 30, 40, 50, 60, 80, and 100 µg p-nitrophenol. The results were expressed in µg p-nitrophenol g dry soil h^{-1}.

Samples 4622–4729

Samples 4622–4729 were collected from four sites in the Moscow region, Russia: a ~70 year old spruce forest (55.457425 N, 37.173583 E), a ~90 year old spruce forest (55.456788 N, 37.188274 E), a highland meadow (55.462282 N, 37.176968 E), and a riparian meadow (55.461148 N, 37.182097 E). In each site, a

set of 27 samples following the 'triangular scheme' was taken (Figure S1). Soil samples for PLFA were taken using a 5 cm diameter soil corer (5 cm depth). The soil was homogenized and a subsample taken for the analysis (frozen at −20°C). The extraction and analysis were done at the University of Göttingen following the methods described in Susanti, et al. [28].

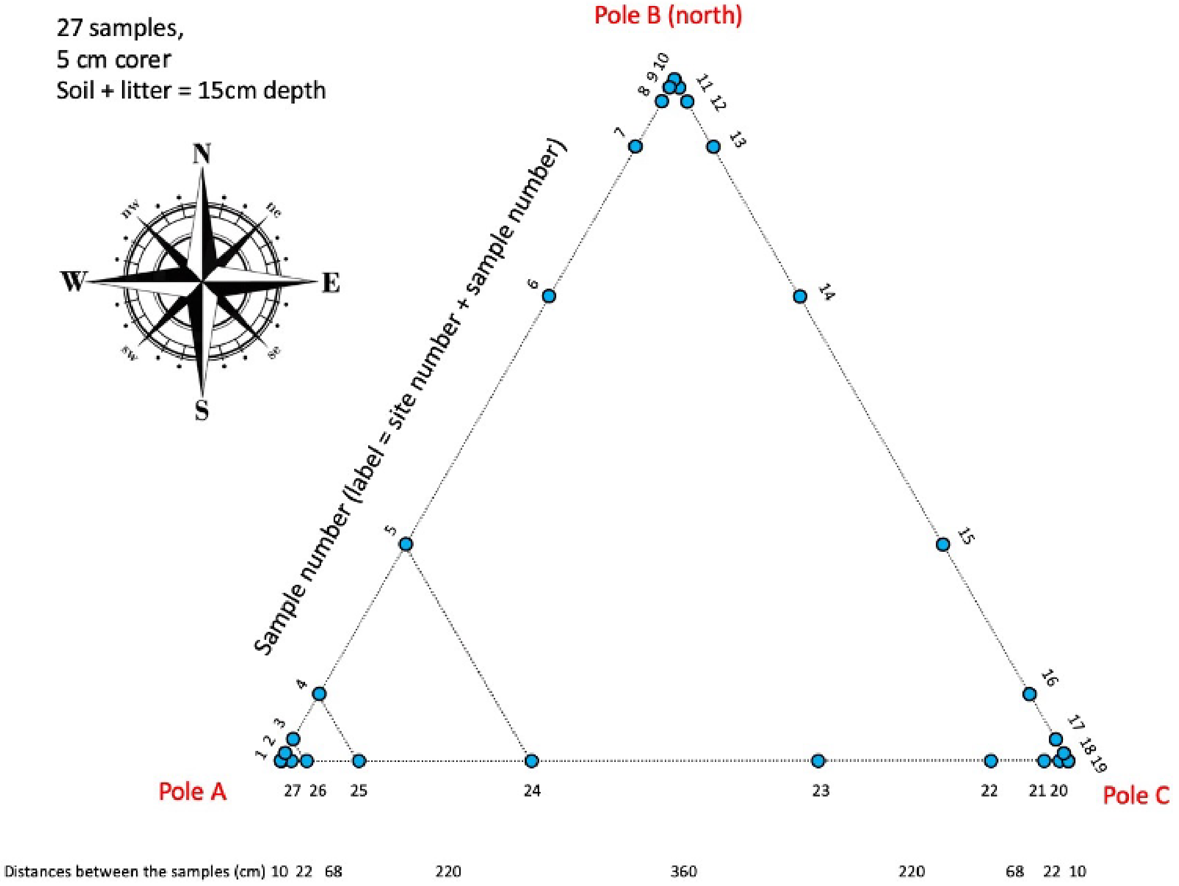

Figure S1: Triangular plot layout of the 27 samples collected at each site.

Samples 6762–6773 and 6908

Soils were sampled at each site as a composite of three subsampling locations spaced at least 25 m apart and situated a minimum of 3 m distance from the nearest tree [29,30]. Representative locations free of visible disturbance or soil erosion were chosen for soil collection. Litter was removed before sampling, and the surface soil (0–5 cm depth) was collected using a trowel in a 30 × 30 cm area. Soils were immediately stored on ice, and transported to the lab for manual homogenization (i.e., mixing of field-moist samples and sieving < 2 mm) prior to enzyme activity assays.

For samples 6762–6773, acid phosphatase, β-glucosidase, and *N*-acetyl-β-glucosaminidase activity assays were based on Tabatabai and Bremner [31] and Tabatabai [27] as modified by Margenot, et al. [32] to account for non-enzymatic contributions to apparent enzyme activity [33,34]. Briefly, 1.0 g oven-dry equivalent of air-dried soil was combined with 5 ml of 10 mM g^{-1} of substrate *p*NP-phosphate (acid phosphatase; pH 6.5), *p*NP-β-glucopyranoside (β-glucosidase; pH 6.0) or *p*NP-*N*-acetyl-β-D-glucosaminide (*N*-acetyl-β-glucosaminidase; pH 5.0) in modified universal buffer (pH 6.5). Mixtures were vortexed to mix and incubated for 1 h at 37°C in a water bath. Assays were alkalized with 4 ml of 0.1 M Tris (pH 12) and 1 ml of 2 M $CaCl_2$. An aliquot of the supernatant was clarified by centrifugation (105 s at 17,968 *g*). *Para*-Nitrophenol product was

quantified by absorbance at 410 nm, and activities were corrected for abiotic hydrolysis of substrate, dissolved organic matter interference, and incomplete product recovery [32,33].

For sample 6908, acid phosphatase activity assays were based on Tabatabai and Bremner [31] as modified by Margenot, et al. [32] to account for non-enzymatic contributions to apparent enzyme activity [33,34]. Briefly, 1.0 g oven-dry equivalent of air-dried soil was combined with 5 ml of 10 mM g^{-1} of *p*NP-phosphate in modified universal buffer (pH 6.5) and incubated for 1 h at 37°C in a water bath. Assays were alkalized with 4 ml of 0.1 M Tris (pH 12) and 1 ml of 2 M $CaCl_2$. An aliquot of the supernatant was clarified by centrifugation (105 s at 17,968 *g*). *Para*-Nitrophenol product was quantified by absorbance at 410 nm, and activities were corrected for abiotic hydrolysis of substrate, dissolved organic matter interference, and incomplete product recovery [32,33].

Samples 7030–7036 and 12175–12178

Permafrost soil samples were collected using sterile metal trowels. For molecular and microbiological analysis, the samples were collected in sterile 250-mL NALGENE boxes. All samples were transported in frozen conditions (−25°C) to Germany. Prior to PFLA extraction, the frozen samples were freeze dried and milled. All samples were subsequently extracted, analysed and the PFLA data were generated as outlined in Mangelsdorf, et al. [35].

Samples 10618–10662

Soil sampling
Samples 10618–10642 were collected from the control plots of a long-term litter manipulation experiment on Gigante Peninsula, Panama, Central America [36]. In each plot, we took 5-8 soil cores (0-10 cm depth) at random locations using a 2 cm (samples 10623–10642) or 3.3 cm diameter (samples 10618–10622) punch corer and combined them to make one composite sample per plot. Upon returning from the field, subsamples were immediately frozen at −50°C for enzyme assays and PLFA analysis. Subsamples for PLFA analysis were subsequently freeze-dried.

Samples 10643–10662 were collected in the control plots of a large-scale comparative experiment in temperate mixed deciduous woodland at Wytham Woods in the UK and in lowland tropical forest in Panama, Central America [37]. On each sampling date we collected five to six soil cores (3.3 cm diameter; 0-10 cm depth) at random locations in each plot. Individual cores were thoroughly mixed to provide one composite sample for each plot. Upon returning from the field, subsamples were immediately frozen at −50°C for enzyme assays and PLFA analysis. Subsamples for PLFA analysis were subsequently freeze-dried.

Enzyme assay methods
For samples 10623–10642, we measured the activity of four hydrolytic enzymes involved in cellulose decomposition and nitrogen- or phosphorus acquisition: Cellobiohydrolase (CBH; EC 3.2.1.91); β-1,4-glucosidase (BG; EC 3.2.1.21); β-1,4-N-acetylglucosaminidase (NAG; EC 3.1.1.14); and acid phosphatase (AP; EC 3.1.3.2). Soil exo-enzyme activity was measured fluorimetrically on 1-g subsamples according to Saiya-Cork, et al. [1] using methylumbelliferone (MUB) labeled substrates in 96-well microplates. Briefly, samples were homogenised in 125 ml sodium acetate buffer (50 mmol L^{-1}, pH 5.0) and pipetted into microplates. Each assay consisted of 16 sample wells (sample solution + substrate), eight blanks (sample solution + buffer), eight negative controls (substrate + buffer), and eight quench standards (standard + sample solution). Prepared plates were incubated in the dark at 20°C for 0.5–3 h depending on the enzyme. Exo-enzyme activity was determined by measuring fluorescence on a microplate reader (BioTek Instruments, Winooski, VT) at 365 nm excitation and 460 nm emission. Fluorescence of the sample wells was corrected

for negative controls, blanks, and quenching; exo-enzyme activity was expressed as substrate conversion over time per g soil (oven-dry weight; μmol h^{-1} g^{-1}).

For samples 10643–10662, hydrolytic soil exoenzyme activities were measured by fluorogenic methods using methylumbelliferyl (MUB) and 7-amino-4-methylcoumarin (AMC). Enzyme assays were performed according to Puissant, et al. [38] and following German, et al. [39] recommendations for measuring enzyme activity in soil solution. For each sample, a soil slurry was prepared by adding 20 mL deionized water to 1 g of soil (fresh weight), then rotary shaking on a magnetic plate for 20 min at 28°C. Enzyme reactions were measured in 96-well microplates containing 50 µL of the specific buffer (25 mM), 50 µL of soil slurry and 100 µL of substrate solution (saturated concentration, 300 µM). Microplates were then incubated in the dark for 3 h at 28°C, with one fluorometric measurement every 30 min (BioSpa 8 Automated Incubator) to follow the kinetics of the reaction. For each sample, three methodological replicates (sample + buffer + substrate) and a quenched standard (sample + buffer + 4-MUB or 7-AMC) were used. Quenching curves were prepared with a serial dilution of 4-MUB solution for different amounts of fluorophore in the well (3000, 2000, 1000 pmol)[38]. For each substrate, a control including the 4-MUB- or 7- AMC-linked substrate and the buffer solution alone were used to check the evolution of fluorescence without enzyme degradation over the duration of assay. The fluorescence intensity was measured using a Cytation 5 spectrophotometer (Biotek) linked to the automated incubator (Biospa 8, Biotek) and set to 330 and 342 nm for excitation and 450 and 440 nm for emission for the 4-MUB and the 7-AMC substrate, respectively.

PLFA assay methods
For samples 10618–10622, phospho-lipid fatty acids (PLFAs) were extracted following the procedure described in Bardgett, et al. [19] and analysed on a Hewlett-Packard 5890 II gas chromatograph equipped with a 5972A mass selective detector (MSD II).

For samples 10643–10652, phospho-lipid fatty acids (PLFAs) were extracted from *c*. 1g freeze-dried soil at a commercial laboratory following the high-throughput method of Buyer and Sasser [40]. Extracts were analysed by gas chromatography (Agilent Series II 6890) and peaks were identified using the Sherlock 6.2™ Microbial Identification System (MIDI, Newark, DE, USA).

Samples 10663–10668

Soil samples 10663–10668 were collected in September 2019 from three 100 × 100 m plots established at each study site in a semi-arid grassland within the Songnen region, Jilin Province, China. Soils were sampled at each plot as a composite of three subsampling locations spaced at least 25 m apart. Litter was removed before sampling, and the surface soil (0–10 cm depth) was collected using a trowel. Soils were immediately stored on ice, and transported to the lab for manual homogenization (i.e., mixing of field-moist samples and sieving < 2 mm) prior to enzyme activity assays.

PLFA extraction for all samples followed the protocol of Frostegård, et al. [17] using 5 g of soil. Lipids were fractionated on silica acid columns (0.5 g silicic acid, 3 mL). Fatty acid methyl esters (FAMEs) were identified based on chromatographic retention times compared with a standard mixture containing 37 FAMEs ranging from C11 to C24. Gas chromatographic analysis was performed on an AutoSystem XL equipped with a HP-5 capillary column (50 m × 0.2 mm inner diameter, film thickness 0.33 µm). The temperature program began at 70°C (held for 2 minutes), increased at 30°C/min to 160°C, then at 3°C/min to 280°C, where it was held for 15 minutes. The injector temperature was set to 260°C, and helium was used as the carrier gas. Individual PLFAs were expressed as a percentage of total PLFAs (relative nmol g^{-1} dry soil weight).

Samples 11361–11364

In August 2019, we collected soil samples using a 2 cm diameter soil drill in the alpine meadow of Nagqu Prefecture, Tibet, China. Soil was stored at −20°C after collection.

Soil PLFA was extracted from 3.0 g freeze-dried soil using a modified version of the Bligh and Dyer method [6]. Briefly, soil samples were extracted twice with 22.8 ml one-phase chloroform-methanol-citrate buffer (0.15 M, pH 4.0); the volume ratio of the mixture was 1:2:0.8. The phospholipids were separated from the neutral lipid and glycolipids on a silicic acid column [41] and were methylated using a mild alkaline methanolysis to derivatize them to their corresponding fatty acid methyl esters (FAMES) [20]. Methyl nonadecanoate fatty acid (19:0) was added as an internal standard. Finally, the FAMEs were dissolved in 150 μ l n-hexane.

For all enzyme assays, controls were included without substrate and without soil samples. Extracellular enzyme activities of all the soils were measured using fluorogenically labelled substrates (Table S1).

Table S1: List of the enzymes along with substrates and associated standards and buffers. MES-buffer represents $C_6H_{13}NO_4SNa_{0.5}$.

Enzyme	Buffers	Substrate	Substrate concentration gradient
β-D-Glucosidase	MES	4-Methylumbelliferyl-β-D-Glucoside	200 µmol
Cellobiosidase	MES	4-Methylumbelliferyl-β-D-Cellobioside	200 µmol
N-acetyl-β-glucosaminidase	MES	4-Methylumbelliferyl-N-acetyl-β-glucosaminide	200 µmol
Phosphatase	MES	4-Methylumbelliferyl-Phosphate	200 µmol
Sulfatase	MES	4-Methylumbelliferyl-Suphate	200 µmol

References

1 Saiya-Cork, K. R., Sinsabaugh, R. L. & Zak, D. R. The effects of long term nitrogen deposition on extracellular enzyme activity in an *Acer saccharum* forest soil. *Soil Biology and Biochemistry* **34**, 1309-1315 (2002). https://doi.org:https://doi.org/10.1016/S0038-0717(02)00074-3

2 Jackson, C. R., Tyler, H. L. & Millar, J. J. Determination of Microbial Extracellular Enzyme Activity in Waters, Soils, and Sediments using High Throughput Microplate Assays. *Journal of Visualized Experiments* **80**, e50399 (2013). https://doi.org:doi:10.3791/50399

3 Bligh, E. G. & Dyer, W. J. A rapid method of total lipid extraction and purification. *Canadian Journal of Biochemistry and Physiology* **37**, 911-917 (1959). https://doi.org:10.1139/o59-099 %M 13671378

4 Frostegård, A. & Bååth, E. The use of phospholipid fatty acid analysis to estimate bacterial and fungal biomass in soil. *Biology and Fertility of Soils* **22**, 59-65 (1996). https://doi.org:10.1007/BF00384433

5 van Aarle, I. M. & Olsson, P. A. Fungal lipid accumulation and development of mycelial structures by two arbuscular mycorrhizal fungi. *Applied and Environmental Microbiology* **69**, 6762-6767 (2003). https://doi.org:doi:10.1128/AEM.69.11.6762-6767.2003

6 Frostegård, Å., Tunlid, A. & Bååth, E. Phospholipid fatty acid composition, biomass, and activity of microbial communities from two soil types experimentally exposed to different heavy metals. *Applied and Environmental Microbiology* **59**, 3605-3617 (1993). https://doi.org:doi:10.1128/aem.59.11.3605-3617.1993

7 Muñoz-Rojas, M., Erickson, T. E., Martini, D., Dixon, K. W. & Merritt, D. J. Soil physicochemical and microbiological indicators of short, medium and long term post-fire recovery in semi-arid ecosystems. *Ecological Indicators* **63**, 14-22 (2016). https://doi.org:https://doi.org/10.1016/j.ecolind.2015.11.038

8 Buyer, J. S., Teasdale, J. R., Roberts, D. P., Zasada, I. A. & Maul, J. E. Factors affecting soil microbial community structure in tomato cropping systems. *Soil Biology and Biochemistry* **42**, 831-841 (2010). https://doi.org:https://doi.org/10.1016/j.soilbio.2010.01.020

9 Borer, E. T. *et al.* Herbivores and nutrients control grassland plant diversity via light limitation. *Nature* **508**, 517-520 (2014). https://doi.org:10.1038/nature13144

10 Siebert, J. *et al.* Drivers of soil microbial and detritivore activity across global grasslands. *Communications Biology* **6**, 1220 (2023). https://doi.org:10.1038/s42003-023-05607-2

11 Maestre, F. T. *et al.* Grazing and ecosystem service delivery in global drylands. *Science* **378**, 915-920 (2022). https://doi.org:doi:10.1126/science.abq4062

12 Frostegård, Å., Tunlid, A. & Bååth, E. Microbial biomass measured as total lipid phosphate in soils of different organic content. *Journal of Microbiological Methods* **14**, 151-163 (1991). https://doi.org:https://doi.org/10.1016/0167-7012(91)90018-L

13 IUSS Working Group. World Reference Base for soil resources 2014, update 2015 international soil classification system for naming soils and creating legends for soil maps. FAO, Rome. (2015).

14 Hallama, M. *et al.* Interactions between cover crops and soil microorganisms increase phosphorus availability in conservation agriculture. *Plant and Soil* **463**, 307-328 (2021). https://doi.org:10.1007/s11104-021-04897-x

15 Marx, M. C., Wood, M. & Jarvis, S. C. A microplate fluorimetric assay for the study of enzyme diversity in soils. *Soil Biology and Biochemistry* **33**, 1633-1640 (2001). https://doi.org:https://doi.org/10.1016/S0038-0717(01)00079-7

16 Poll, C., Ingwersen, J., Stemmer, M., Gerzabek, M. H. & Kandeler, E. Mechanisms of solute transport affect small-scale abundance and function of soil microorganisms in the detritusphere. *European Journal of Soil Science* **57**, 583-595 (2006). https://doi.org:https://doi.org/10.1111/j.1365-2389.2006.00835.x

17 Frostegård, Å., Bååth, E. & Tunlio, A. Shifts in the structure of soil microbial communities in limed forests as revealed by phospholipid fatty acid analysis. *Soil Biology and Biochemistry* **25**, 723-730 (1993).

18 Kramer, S., Marhan, S., Haslwimmer, H., Ruess, L. & Kandeler, E. Temporal variation in surface and subsoil abundance and function of the soil microbial community in an arable soil. *Soil Biology and Biochemistry* **61**, 76-85 (2013). https://doi.org:https://doi.org/10.1016/j.soilbio.2013.02.006

19 Bardgett, R. D., Hobbs, P. J. & Frostegård, Å. Changes in soil fungal:bacterial biomass ratios following reductions in the intensity of management of an upland grassland. *Biology and Fertility of Soils* **22**, 261-264 (1996). https://doi.org:10.1007/BF00382522
20 White, D. C., Davis, W. M., Nickels, J. S., King, J. D. & Bobbie, R. J. Determination of the sedimentary microbial biomass by extractible lipid phosphate. *Oecologia* **40**, 51-62 (1979). https://doi.org:10.1007/BF00388810
21 Drescher, J. *et al.* Ecological and socio-economic functions across tropical land use systems after rainforest conversion. *Philosophical Transactions of the Royal Society B: Biological Sciences* **371**, 20150275 (2016). https://doi.org:doi:10.1098/rstb.2015.0275
22 Krashevska, V., Sandmann, D., Maraun, M. & Scheu, S. Moderate changes in nutrient input alter tropical microbial and protist communities and belowground linkages. *The ISME Journal* **8**, 1126-1134 (2014). https://doi.org:10.1038/ismej.2013.209
23 Casida, L. E. J., Klein, D. A. & Santoro, T. Soil Dehydrogenase Activity. *Soil Science* **98**, 371-376 (1964).
24 Kandeler, E. & Gerber, H. Short-term assay of soil urease activity using colorimetric determination of ammonium. *Biology and Fertility of Soils* **6**, 68-72 (1988). https://doi.org:10.1007/BF00257924
25 Huising, E. J. *et al.* in *Manual de biologia dos solos tropicais: amostragem e caracterização da biodiversidade* (eds F.M.S. Moreira, E.J. Huising, & D.E. Bignell) 43-77 (UFLA, 2010).
26 Swift, M. & Bignell, D. E. Standard methods for the assessment of soil biodiversity and land-use-practice. International Centre for Research in Agroforestry, South East Asian Regional Research Programme, ASB-Lecture Note 6B, Bogor, Indonesia. (2001).
27 Tabatabai, M. A. in *Methods of Soil Analysis* (eds R.W. Weaver *et al.*) 775-833 (Soil Science Society of America, 1994).
28 Susanti, W. I., Pollierer, M. M., Widyastuti, R., Scheu, S. & Potapov, A. Conversion of rainforest to oil palm and rubber plantations alters energy channels in soil food webs. *Ecology and Evolution* **9**, 9027-9039 (2019). https://doi.org:https://doi.org/10.1002/ece3.5449
29 Gu, C., Wilson, S. G. & Margenot, A. J. Lithological and bioclimatic impacts on soil phosphatase activities in California temperate forests. *Soil Biology and Biochemistry* **141**, 107633 (2020). https://doi.org:https://doi.org/10.1016/j.soilbio.2019.107633
30 Wilson, S. G., Dahlgren, R. A., Margenot, A. J., Rasmussen, C. & O'Geen, A. T. Expanding the Paradigm: The influence of climate and lithology on soil phosphorus. *Geoderma* **421**, 115809 (2022). https://doi.org:https://doi.org/10.1016/j.geoderma.2022.115809
31 Tabatabai, M. A. & Bremner, J. M. Use of p-nitrophenyl phosphate for assay of soil phosphatase activity. *Soil Biology and Biochemistry* **1**, 301-307 (1969). https://doi.org:https://doi.org/10.1016/0038-0717(69)90012-1
32 Margenot, A. J., Nakayama, Y. & Parikh, S. J. Methodological recommendations for optimizing assays of enzyme activities in soil samples. *Soil Biology and Biochemistry* **125**, 350-360 (2018). https://doi.org:https://doi.org/10.1016/j.soilbio.2017.11.006
33 Daughtridge, R. C., Nakayama, Y. & Margenot, A. J. Sources of abiotic hydrolysis of chromogenic substrates in soil enzyme assays: Storage, termination, and incubation. *Soil Biology and Biochemistry* **158**, 108245 (2021). https://doi.org:https://doi.org/10.1016/j.soilbio.2021.108245
34 Nakayama, Y., Wade, J., Li, C., Daughtridge, R. C. & Margenot, A. J. Quantifying the relative importance of controls and assay conditions for reliable measurement of soil enzyme activities with para-nitrophenol substrates. *Geoderma* **429**, 116234 (2023). https://doi.org:https://doi.org/10.1016/j.geoderma.2022.116234
35 Mangelsdorf, K., Bajerski, F., Karger, C. & Wagner, D. Identification of a novel fatty acid in the cell membrane of *Chryseobacterium frigidisoli* PB4T isolated from an East Antarctic glacier forefield. *Organic Geochemistry* **106**, 68-75 (2017). https://doi.org:https://doi.org/10.1016/j.orggeochem.2017.01.003
36 Sayer, E. J. *et al.* Tropical forest above-ground productivity is maintained by nutrients cycled in litter. *Journal of Ecology* **112**, 690-700 (2024). https://doi.org:https://doi.org/10.1111/1365-2745.14251

37 Bréchet, L. M. *et al.* Distinct responses of soil respiration to experimental litter manipulation in temperate woodland and tropical forest. *Ecology and Evolution* **8**, 3787-3796 (2018). https://doi.org:https://doi.org/10.1002/ece3.3945
38 Puissant, J. *et al.* Seasonal influence of climate manipulation on microbial community structure and function in mountain soils. *Soil Biology and Biochemistry* **80**, 296-305 (2015). https://doi.org:https://doi.org/10.1016/j.soilbio.2014.10.013
39 German, D. P. *et al.* Optimization of hydrolytic and oxidative enzyme methods for ecosystem studies. *Soil Biology and Biochemistry* **43**, 1387-1397 (2011). https://doi.org:https://doi.org/10.1016/j.soilbio.2011.03.017
40 Buyer, J. S. & Sasser, M. High throughput phospholipid fatty acid analysis of soils. *Applied Soil Ecology* **61**, 127-130 (2012). https://doi.org:https://doi.org/10.1016/j.apsoil.2012.06.005
41 Gehron, M. J. & David C, W. Sensitive assay of phospholipid glycerol in environmental samples. *Journal of Microbiological Methods* **1**, 23-32 (1983). https://doi.org:https://doi.org/10.1016/0167-7012(83)90004-0
